# Caspase-3 Levels Alter With Non-surgical Periodontal Therapy in Patients With Periodontitis

**DOI:** 10.7759/cureus.54119

**Published:** 2024-02-13

**Authors:** Santo G Umesh, Sankari Malaiappan

**Affiliations:** 1 Periodontics, SRM Dental College Ramapuram, Chennai, IND; 2 Periodontics, Saveetha Dental College and Hospitals, Saveetha Institute of Medical and Technical Sciences, Saveetha University, Chennai, IND

**Keywords:** non-surgical periodontal therapy, biomarker, periodontitis, apoptotic factors, caspase-3

## Abstract

Background

This study aims to evaluate the levels of caspase-3 in the gingival crevicular fluid (GCF) of chronic periodontitis patients before and after phase I treatment and compare it with those of healthy controls.

Methodology

The study involved 40 participants who were divided into two groups. Group 1 consisted of 30 chronic periodontitis patients, and group 2 consisted of 10 healthy controls. GCF was collected at baseline for both groups and at three months for group 1. Periodontal parameters and caspase-3 levels were analyzed before and after non-surgical therapy.

Results

Caspase-3 levels were higher in patients with chronic periodontitis compared with healthy controls. However, comparing baseline and postoperative levels, there was a statistically significant reduction in periodontal parameters and caspase-3 levels, with 0.80 ± 0.03 at baseline and 0.44 ± 0.02 at three months after non-surgical periodontal therapy.

Conclusions

Caspase-3, being the key molecule in apoptosis, was found to be at lower concentrations in healthy gingiva and was increased in the presence of periodontal disease. However, with non-surgical periodontal therapy, caspase-3 levels decreased, proving that non-surgical periodontal therapy affects host immune mechanisms and reduces apoptosis, thereby preventing disease progression.

## Introduction

Periodontitis is a multifactorial disease that results from the interaction between the pathogenic organisms and host responses to pathogenic invasion. The destruction of the periodontal tissues may be either due to the pathogen invasion or host immune responses. The destruction by the pathogen comprises virulence factors that majorly trigger host immune responses. Host destructive mechanisms may be due to inflammatory responses characterized by the activity of neutrophils, lymphocytes, macrophages, and other inflammatory cells. During this process, pathogenic organisms along with host tissues are affected. The degradation of host tissues leads to disease progression. However, the exact pathogenesis of periodontitis remains unclear due to its varied etiologic factors and diverse destructive mechanisms [1,2].

The pathogenesis may be directly modulated by bacteria which affects the periodontal tissues. *Porphyromonas gingivalis*, *Prevotella loescheii*, and *Fusobacterium nucleatum* contain carboxylic acid chains that induce apoptosis in T cells and gingival keratinocytes [3-5]. *Aggregatibacter actinomycetemcomitans* releases a toxin that induces apoptosis in B lymphocytes [6]. However, apoptosis of neutrophils can be delayed by phagocytosis of bacteria due to exposure to bacterial components, such as lipopolysaccharides [7]. Growing evidence suggests that the pathogenesis of periodontal diseases may be induced by apoptosis [8].

Apoptosis, or programmed cell death, is central to the development and homeostasis of metazoans [9]. Dysregulation of apoptosis leads to various human pathologies, including cancer, autoimmune diseases, and neurodegenerative disorders [10]. Since the concept of apoptosis was established in 1972, research efforts have led to the identification of hundreds of genes that control the initiation, execution, and regulation of apoptosis in several species [11]. Apoptosis plays an important role in tissue homeostasis in multicellular organisms, and its disruption has been associated with several disorders, such as cancer, acquired immunodeficiency syndrome, Alzheimer’s disease, rheumatoid arthritis, and periodontal diseases [12,13]. Compelling evidence shows that the mechanism of apoptosis is evolutionarily conserved.

The core elements of the apoptotic response are caspases. The conserved family of enzymes known as caspases (cysteine proteases that cleave after an aspartate residue in their substrates) irreversibly causes a cell to die. The recognized functions of caspases in apoptosis (caspases 3, 6, 7, 8, and 9 in mammals) and inflammation (caspases 1, 4, 5, and 12 in humans and caspases 1, 11, and 12 in mice) have led to a general classification of caspases. Caspases 2 and 10 have a more difficult-to-classify function. Initiator caspases (caspases 8 and 9) and executioner caspases (caspases 3, 6, and 7) are the two subclasses of caspases that are involved in apoptosis [14]. The prodomains of several caspases have various protein-protein interaction domains, enabling them to aggregate with various adapters. For instance, although caspases 8 and 10 include a death effector domain, caspases 1, 2, 4, 5, and 9 contain a caspase recruitment domain [15].

Caspase-3 acts as an executioner caspase and plays a major role in apoptosis. The pathogenesis of periodontal diseases comprises both invasion and defensive mechanisms, and cell death due to both activities is common and highly expected. The pathogenic role of caspase-3 in periodontal diseases has been proven, with caspase 3 levels increased in periodontal diseases [16]. However, the effect of non-surgical periodontal therapy on caspase-3 levels has not been studied so far. Hence, this study was undertaken to compare caspase-3 levels of periodontitis patients before and after non-surgical periodontal therapy.

## Materials and methods

Study population

The study protocol was approved by the scientific review board of the university (SRB approval number: SRB/SD/MDS16PER/01) as well as by the Institutional Human Ethical Committee. This study was registered with the Clinical Trial Registry of India (registration number: CTRI/2018/06/014600). The study objectives were explained to all patients, and written informed consent was obtained from all participants before including them in the study. A sample size of 28 was derived with a power calculation of 95%. Study participants were recruited from the outpatient department based on the inclusion and exclusion criteria for periodontitis, as shown in Table 1.

**Table 1:** Inclusion and exclusion criteria.

Inclusion criteria	Exclusion criteria
Patients diagnosed clinically as having chronic periodontitis according to the American Academy of Periodontology criteria 1999	Patients with a history of any systemic diseases such as diabetes mellitus and cardiac problems
Patients aged between 28 and 70 years	Patients with a previous history of hospitalization/surgery in the past one year
	Pregnant or lactating women
	Patients with a history of antibiotic or anti-inflammatory drug intake in the past six months
	Patients on treatment with steroids
	Patients with congenital abnormalities

Study groups

Study participants were divided into the following two groups: group 1: patients with generalized chronic periodontitis (preoperative); and group 2: patients with generalized chronic periodontitis (postoperative, three months after scaling and root planing).

At a study power of 95%, the sample size was calculated using G’Power 3.1 software. Power calculation was performed using the data from a similar study. A total of 28 patients were required to achieve a significant difference between the two groups. Hence, for the present study protocol, 30 patients would be sufficient to achieve the required significance at 95% power with an alpha error set at 0.05.

Assessment of clinical and biochemical parameters

Parameters were assessed before and after non-surgical periodontal therapy. Non-surgical therapy included scaling and root planing followed by oral hygiene instructions. Scaling was done using ultrasonic scalers. Root planing was done using Gracey curettes.

Gingival Bleeding Index

The gingival bleeding index [17] was measured by probing the gingival crevice. The degree of bleeding was scored and the average was calculated. Gingival bleeding recordings were made using a mouth mirror under illumination. The bleeding was assessed by probing gently in the mesial and distal facial papilla and the lingual marginal gingiva.

Probing Pocket Depth

The probing depth was measured at six sites from the gingival margin until the base of the gingival sulcus for all teeth and the average was calculated. The pocket depth was measured from the crest of the gingival margin to the base of the pocket using a pressure-sensitive UNC-15 probe with markings from 0 to 15 with bands at 5, 10, and 15 mm.

Clinical Attachment Level

The clinical attachment level was measured at six sites per tooth from the cementoenamel junction until the base of the gingival sulcus. Clinical attachment level was measured using a pressure-sensitive UNC-15 probe from the cementoenamel junction to the base of the periodontal pocket.

Assessment of gingival crevicular fluid caspase-3 level

Gingival Crevicular Fluid Collection

The clinical examination and site selection for sample procurement were done by a single examiner. After making the participant sit comfortably in an upright position on the dental chair, the tooth of interest was selected based on probing depth. The site was first isolated using cotton rolls, and the supra-gingival plaque was removed using scalers without disturbing the gingival margin to avoid contamination and blocking of the capillary pipette. For patients allocated to group 1, gingival crevicular fluid (GCF) was collected using 1-5 µL calibrated volumetric microcapillary tubes (SIGMA Aldrich Chemical Company, USA) by placing it at the entrance of the gingival sulcus for 5-20 minutes and gently touching the gingival margin. A standardized volume of 1 µL of GCF was collected from the test site in the periodontitis groups. The test sites that did not express the standard volume of GCF (1 µL) and sites with blood and saliva contamination were excluded. Following collection, the samples were diluted with 99 µL of phosphate-buffered saline in Eppendorf tubes and stored at -20°C until the assay procedure was performed.

Once the GCF was collected, the patients underwent the following treatment. All patients included in group 1 underwent scaling and root planing. Scaling was performed using ultrasonic scalers and root planing by site-specific Gracey Curettes. The site was cleared of any blood and dried. Patients were asked not to rinse their mouths or consume food for one hour. They were recalled after three months for assessing clinical parameters and GCF collection.

Caspase-3 Detection in Gingival Crevicular Fluid by Enzyme-Linked Immunosorbent Assay

Using a pre-coated enzyme-linked immunosorbent assay plate and following the manufacturer’s instructions (RayBiotech, Inc., Georgia), the total caspase-3 levels were ascertained. In summary, 50 µL of reference solutions or samples were added to the corresponding wells in duplicate. The plates were then rinsed four times with 1× buffer and allowed to incubate for two hours at room temperature with constant shaking (200 rpm). After adding 100 µL of human caspase-3 detection antibody solution to each well and shaking the plate for an hour at room temperature, the plates were once again cleaned with 1× buffer. After adding 100 µL of Avidin-HRP D solution to each well and mixing it for 30 minutes at room temperature, the plate was cleaned using 1× buffer. Following a 30-minute dark incubation period, 100 µL of substrate solution F was applied to each well. By adding 100 µL of stop solution to each well, the reaction was halted, and the color of each well changed from blue to yellow. After 30 minutes, the absorbance was measured at 450 nm using a BioReader™.

## Results

The mean ± standard deviation of probing depth in healthy controls was 2.04 ± 0.24 mm. The caspase-3 level among healthy controls was 0.23 ± 0.03 (pg/mL) (Table 2).

**Table 2:** Clinical and biochemical parameters of healthy controls.

Parameters	Healthy controls
Bleeding on probing	0
Probing depth (mm)	2.04 ± 0.24
Clinical attachment level (mm)	0
Caspase-3 levels (pg/mL)	0.23 ± 0.03

On comparing the bleeding on probing levels, the mean ± standard deviation was 1.63 ± 0.22 and 0.58 ± 0.26 pretreatment and three months after non-surgical periodontal therapy, respectively. This difference was highly statistically significant (p = 0.00) Comparison of the probing depth levels showed a statistically significant difference with a mean ± standard deviation of 7.02 ± 0.49 mm before treatment and 5.27 and 0.58 mm after non-surgical periodontal therapy (p = 0.00) (Table 3).

**Table 3:** Comparison of clinical parameters among chronic periodontitis patients before and after NST. *: significant at <0.05. NST = non-surgical therapy

Parameters	Before NST	After NST	P-value
Bleeding on probing	1.49 ± 0.24	0.55 ± 0.14	0.00*
Probing depth (mm)	5.21 ± 0.51	3.81 ± 0.49	0.00*
Clinical attachment level (mm)	6.14 ± 0.57	4.73 ± 0.54	0.00*

**Figure 1:**
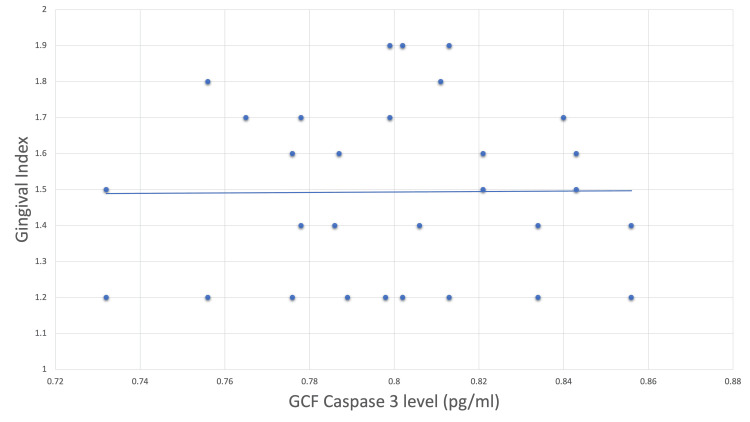
Scatter plot correlating baseline caspase-3 levels with gingival index. GCF = gingival crevicular fluid

The correlation of baseline caspase-3 levels with the gingival index revealed a positive correlation between the two values with an r-value of 0.240 (Figure 1).

**Figure 2:**
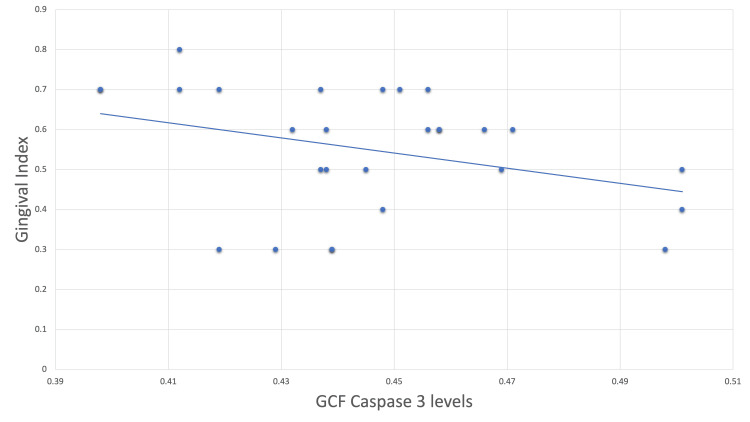
Scatter plot correlating postoperative caspase-3 levels with gingival index. GCF = gingival crevicular fluid

However, the postoperative correlation of caspase-3 levels with the gingival index revealed a negative correlation with an r-value of -0.115 (Figure 2).

**Figure 3:**
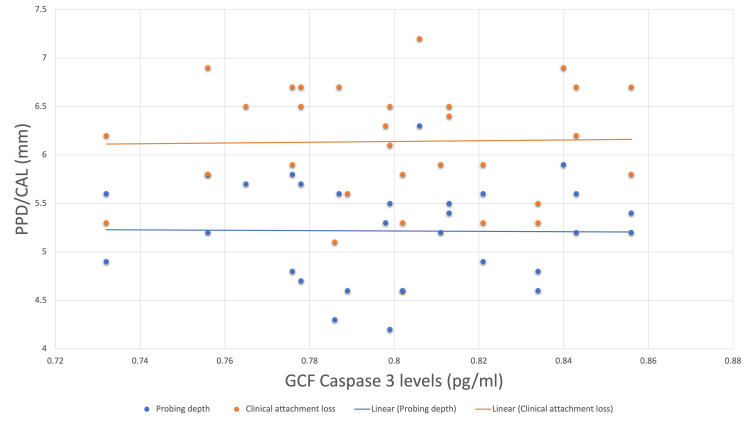
Scatter plot correlating baseline caspase-3 levels with probing depth and clinical attachment loss. PPD = probing pocket depth; CAL = clinical attachment loss; GCF = gingival crevicular fluid

**Figure 4:**
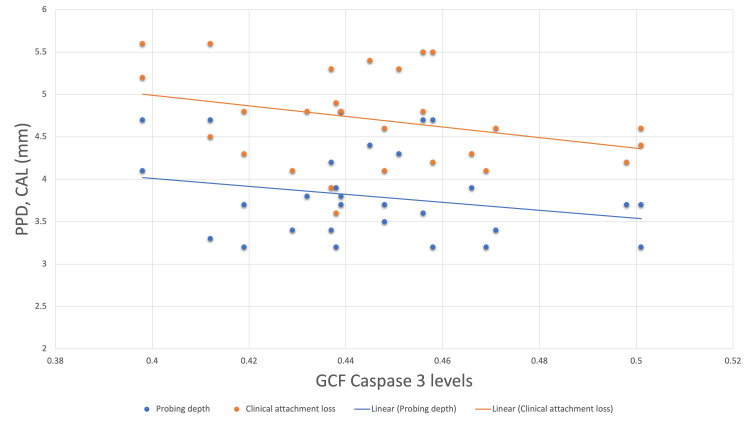
Scatter plot correlating postoperative caspase-3 levels with probing depth and clinical attachment loss. PPD = probing pocket depth; CAL = clinical attachment loss; GCF = gingival crevicular fluid

Correlation of baseline and postoperative caspase-3 levels with probing depth revealed a weak negative correlation with an r-value of -0.170 and -0.145, respectively. However, both values did not show statistically significant differences. On correlating baseline caspase-3 levels with clinical attachment levels, a mild positive correlation was found between the two values with an r-value of 0.106. A weak negative correlation with an r-value of -0.007 was found when correlating postoperative caspase-3 levels with clinical attachment level (Figures 3, 4).

However, both values did not show statistically significant differences (Table 4).

**Table 4:** Correlation of caspase-3 levels with clinical parameters. *: significant at <0.05. NST = non-surgical therapy

Clinical parameters	Statistical test	Caspase-3 level	
		Before NST	After NST
Bleeding on probing	r-value	0.009	-0.334
	p-value	0.964	0.07
Probing depth	r-value	-0.012	-0.215
	p-value	0.951	0.253
Clinical attachment level	r-value	0.022	-0.249
	p-value	0.907	0.184

## Discussion

Periodontitis is a multifactorial disease that results from the interaction between the pathogenic organisms and host responses to pathogenic invasion. The destruction of the periodontal tissues may be either due to pathogen invasion or due to host immune responses. The destruction by the pathogen comprises virulence factors that majorly trigger host immune responses. Growing evidence suggests that the pathogenesis of periodontal diseases may also be induced by apoptosis [8,18,19]. Caspases are the central components of the apoptotic response [20-22]. Caspases (which are so-named as they are cysteine proteases that cleave after an aspartate residue in their substrates) are a conserved family of enzymes that irreversibly commit a cell to die [23]. Therefore, this study was undertaken to know the effect of non-surgical periodontal therapy on caspase-3 levels.

In this study, there was a statistically significant reduction in caspase-3 levels after non-surgical periodontal therapy. The mean ± standard deviation of GCF caspase-3 levels at baseline was 0.80 ± 0.03 pg/mL and after non-surgical periodontal therapy was 0.44 ± 0.02 pg/mL. This indicated that apoptosis had reduced after periodontal therapy, thereby proving that periodontal treatment has a positive effect on apoptosis and host defense mechanisms.

Although caspase-3 levels were reduced after non-surgical periodontal therapy, the clinical parameters did not positively correlate with caspase-3 levels. The possible reason for this negative correlation could be due to the follow-up period of three months after non-surgical periodontal therapy. The other reason could be that surgical intervention was not done and therefore complete reversal of disease was not possible.

Tissue caspase-3 levels were estimated in a previous study that compared quantities of immunohistochemically identified p53, Bcl-2, and caspase-3 in gingival tissue from patients with generalized aggressive periodontitis (caspase-3: grade 0: 50%) and healthy subjects (caspase-3: Grade 0: 60%) [24]. Gingival biopsies were procured from patients with periodontitis undergoing flap surgery and healthy patients undergoing third molar extractions. The study found no significant differences between these groups concerning the overall frequency of caspase-3. Another similar study [25] determined the differences in apoptosis and caspase-3 expression in patients with plaque-induced gingivitis (caspase-3: 100%) compared to patients with cyclosporin-induced gingival overgrowth (grade 0: 68% and grade 1: 32%). The study observed that the extent of keratinocyte apoptosis and the decreased level of caspase-3 may play an important role in the gingiva of kidney transplant recipients with cyclosporin-induced gingival overgrowth. As the samples were gingival tissues and the results were expressed as grades, a comparison could not be done with the present study.

Kayagaki et al. [26] studied the expression of apoptotic factors in inflamed and non-inflamed gingival overgrowth tissues. Apoptotic index, caspase-3 expression (Inflamed tissue: 8.3 ± 2.0 and non-inflamed tissue: 7.3 ± 1.9), the proliferative index, FOXO1 expression, and histological inflammation were measured in situ. Analysis of data showed that apoptosis decreased in all forms of gingival overgrowth examined, and inflammation caused a small but significant increase compared with non-inflamed tissues. The least apoptosis occurred in the most fibrotic tissues. Cell proliferation was elevated in all forms of gingival overgrowth tested, independent of inflammation. Comparisons of inflamed and non-inflamed sites revealed a trend toward increased caspase-3 expression with inflammation, but the difference between caspase levels as a function of inflammation was not statistically significant. Although the methodology of this study was not similar to the present study, the results were comparable. In the presence of inflammation, caspase-3 levels were increased. This is similar to our results where the caspase-3 levels were elevated in periodontitis patients than healthy controls.

In the study by Mandal et al., the expression of caspase-3 in type 2 diabetes patients with periodontitis was assessed. The study compared serum and GCF caspase-3 levels among periodontitis patients with uncontrolled diabetes, periodontitis patients with controlled diabetes, patients only with periodontitis, and healthy controls. The results showed elevated caspase-3 levels in chronic periodontitis patients when compared with healthy controls. Chronic periodontitis patients with uncontrolled diabetes (887 ± 71.4 pg/mL) showed the highest expression of caspase-3 levels when compared with periodontitis patients with controlled diabetes (498 ± 176.6 pg/mL)) and patients with only periodontitis (274 ± 48.45 pg/mL). The GCF caspase-3 level in healthy controls was 165.1 ± 9.07 pg/mL which was similar to our study where the GCF caspase-3 level was 230 ± 3.03 pg/mL [27].

Lee et al. estimated the levels of cleaved caspase-3 in apoptosis in patients with chronic periodontitis and compared them with healthy controls. They used immunostaining methods with caspase-3 antibodies and showed that cleaved caspase-3 levels were elevated in the diseased tissues (group 1: cleaved caspase-3: 1.7 ± 0.2, and group 2: cleaved caspase-3: 0.3 ± 0.2) These differences were statistically significant and, in the case of cleaved caspase-3, highly significant. Although the estimation of caspase levels in both studies was from different types of samples (gingival tissue, GCF), the results were similar to the present study showing that GCF caspase-3 levels increased with disease progression [28].

Roger et al. studied whether sex dimorphism exists in the expression of inflammatory and apoptotic mediators in gingiva obtained from normal and diseased sites of periodontal tissues. Gingival samples were grouped by the sulcus depth from the adjacent teeth as follows: 1-3 mm (normal), 3 mm with bleeding on probing (slight disease), 3-6 mm (moderate disease), and >6 mm (severe disease). The tissue concentrations of caspase-3 were determined by enzyme-linked immunosorbent assay. The concentration of caspase-3 in the gingival tissues was greater in females with the severe form of the disease (136.69 ± 23.3 pg/mL) than in the gingival tissues derived from mild (25.06 ± 5.71 pg/mL) and moderate sites (78.22 ± 19.2 pg/mL). This study also showed that caspase-3 levels increased with disease severity which was in accordance with the present study which showed that caspase-3 levels were elevated in chronic periodontitis patients when compared with healthy controls [29].

In a previous study by Pradeep et al., GCF caspase-3 levels were assessed in healthy individuals, patients with gingivitis, and patients with periodontitis and were found to increase with disease progression. The caspase-3 levels were 160 pg/mL in healthy controls and 960 pg/mL in periodontitis patients. As seen in this study, when compared with healthy controls (230 pg/mL), periodontitis patients had increased GCF caspase-3 levels (870 pg/mL). Because it was a cross-sectional study, postoperative values could not be compared [16].

Our study showed that caspase-3 levels were reduced after non-surgical periodontal therapy. Yet, the changes after surgical management were not evaluated. This is a potential limitation of this study. Another limitation of the study is that caspase-3 is a systemic marker of apoptosis, and, therefore, the alterations in serum samples should also be evaluated to substantiate the fact. Further studies are required to assess the levels of caspase-3 after surgical intervention comparing gingival tissues and GCF levels to establish a definitive role of caspase-3 in the pathogenesis of periodontal disease.

## Conclusions

GCF caspase-3 levels were higher in patients with chronic periodontitis when compared with healthy controls. GCF caspase-3 levels were reduced after non-surgical periodontal therapy. Thus, after non-surgical periodontal therapy, caspase-3 levels decreased, proving that non-surgical periodontal therapy affects host immune mechanisms and reduces apoptosis, thereby preventing disease progression. This result also correlated with clinical parameters which improved on non-surgical periodontal therapy. However, complete recovery from the disease may not be attained with phase I periodontal therapy alone. Further longitudinal studies are required to assess the definitive role of caspase-3 in the pathogenesis of periodontal diseases.
